# Abstinence and Fear Experienced during This Period Produce Distinct Cortical and Hippocampal Adaptations in Alcohol-Dependent Rats

**DOI:** 10.3390/brainsci14050431

**Published:** 2024-04-26

**Authors:** Noah L. Steiner, Dvijen C. Purohit, Casey M. Tiefenthaler, Chitra D. Mandyam

**Affiliations:** 1 VA San Diego Healthcare System, San Diego, CA 92161, USA; nsteiner@ucsd.edu (N.L.S.);; 2 Skaggs School of Pharmacy and Pharmaceutical Sciences, University of California San Diego, San Diego, CA 92093, USA; cmtiefenthaler@health.ucsd.edu; 3 Department of Anesthesiology, University of California San Diego, San Diego, CA 92093, USA

**Keywords:** alcohol dependence, prefrontal cortex, dentate gyrus, GluN, Golgi–Cox, trace fear conditioning

## Abstract

Previous studies demonstrate that ethanol dependence induced by repeating cycles of chronic intermittent ethanol vapor exposure (CIE) followed by protracted abstinence produces significant gray matter damage via myelin dysfunction in the rodent medial prefrontal cortex (mPFC) and alterations in neuronal excitability in the mPFC and the dentate gyrus (DG) of the hippocampus. Specifically, abstinence-induced neuroadaptations have been associated with persistent elevated relapse to drinking. The current study evaluated the effects of forced abstinence for 1 day (d), 7 d, 21 d, and 42 d following seven weeks of CIE on synaptic plasticity proteins in the mPFC and DG. Immunoblotting revealed reduced expression of CaMKII in the mPFC and enhanced expression of GABA_A_ and CaMKII in the DG at the 21 d time point, and the expression of the ratio of GluN2A/2B subunits did not change at any of the time points studied. Furthermore, cognitive performance via Pavlovian trace fear conditioning (TFC) was evaluated in 3 d abstinent rats, as this time point is associated with negative affect. In addition, the expression of the ratio of GluN2A/2B subunits and a 3D structural analysis of neurons in the mPFC and DG were evaluated in 3 d abstinent rats. Behavioral analysis revealed faster acquisition of fear responses and reduced retrieval of fear memories in CIE rats compared to controls. TFC produced hyperplasticity of pyramidal neurons in the mPFC under control conditions and this effect was not evident or blunted in abstinent rats. Neurons in the DG were unaltered. TFC enhanced the GluN2A/2B ratio in the mPFC and reduced the ratio in the DG and was not altered by abstinence. These findings indicate that forced abstinence from CIE produces distinct and divergent alterations in plasticity proteins in the mPFC and DG. Fear learning-induced changes in structural plasticity and proteins contributing to it were more profound in the mPFC during forced abstinence.

## 1. Introduction

Moderate to severe alcohol use disorder (AUD) has been reported to result in cognitive deficits in humans [1,2,3]. Clinical studies have identified the deleterious effects of chronic alcohol exposure in the prefrontal cortex (PFC) and the hippocampus, which has been shown to be particularly sensitive to alcohol-induced damage [3,4,5,6,7,8,9,10,11,12,13,14]. Our lab has previously shown that persistent alcohol exposure produces significant neuroadaptations in the PFC and hippocampus, including alterations in the proliferation and maturation of oligodendrocytes and neuroplasticity in the PFC and the proliferation, maturation, and differentiation of neural cell progenitors and neuroplasticity in the hippocampus [15,16]. However, there is limited understanding regarding whether persistent alcohol exposure produces divergent or comparable neuroadaptations in the PFC and hippocampus during forced abstinence, and whether these adaptations predict the cognitive deficits seen during abstinence.

Pavlovian conditioning is a form of associative learning where a neutral conditional stimulus (CS; e.g., tone) becomes associated with an aversive unconditional stimulus (US; e.g., foot shock). Through pairings of the two stimuli, the CS comes to elicit a conditional response. Trace fear conditioning (TFC) is a procedure where the termination of the CS precedes the onset of the US. That is, a trace interval is imposed between the CS and US; thus, the two stimuli are temporally discontiguous. It has been established that TFC is hampered in animal models of AUD [17,18,19,20,21,22,23]. However, it is unclear if deficits in the consolidation of trace fear memories during abstinence from AUD are paralleled by comparable or divergent glutamatergic neuroadaptations or structural plasticity of neurons in the PFC and the hippocampus.

To answer these questions, our study used the chronic intermittent ethanol vapor exposure (CIE) model, an established animal model of moderate to severe AUD. It implements daily cycles of intoxication via ethanol vapor and withdrawal to induce clinical signs of alcohol dependence, such as somatic withdrawal symptoms and escalated ethanol drinking in rats [24,25]. The rodent medial prefrontal cortex (mPFC) and the dentate gyrus (DG) of the hippocampus are regions that have been shown to be significantly affected as a result of the CIE paradigm [16,26,27,28,29,30,31,32,33,34,35]. Impairments in mPFC and hippocampal function produced by CIE are associated with altered glutamatergic plasticity in these brain regions (e.g., altered neuronal excitability, functional and structural neuronal plasticity, and neurogenesis [15,16,31,36,37,38,39,40,41,42,43,44,45]). Therefore, this study tested the hypothesis that early, mid, and late abstinence from CIE produces similar neuroadaptations in the mPFC and hippocampus, specifically in proteins supporting glutamatergic neurotransmission. This study also tested the subhypothesis that early abstinence-induced effects on the consolidation of trace fear memories would be predicted by similar neuronal adaptations in the mPFC and hippocampus. 

## 2. Methods

### 2.1. Animals

Adult male Wistar rats were housed in a temperature-controlled (22 °C) vivarium with ad libitum access to food and water. Animals were set on a 12 h (hour) light/12 h dark cycle, with the light cycle beginning at 8:00 p.m., in groups of 2 to 3 animals per cage. Experimental procedures were carried out in strict adherence to the NIH Guide for the Care and Use of Laboratory Animals and approved by the Institutional Animal Care and Use Committee of VA San Diego Healthcare System (approval number A16-000). Animals were 7–8 weeks old and weighed 180–200 g at the beginning of the study.

### 2.2. Chronic Intermittent Ethanol Vapor Exposure (CIE)

During CIE, rats in their cages were placed in vapor chambers (animal housing rooms different from the Vivarium). CIE rats were exposed to cycles of ethanol vapor produced via the vaporization of 95% ethanol in a heated flask that was immediately conveyed through controlled air flow to rat vapor chambers on a 14 h on/10 h off daily schedule for the duration of seven weeks. The vapor flow rate was calibrated so as to achieve animal target blood alcohol levels (BALs) within the range of 125 to 250 mg/dL. Blood sampling (tail bleedings) was performed immediately after daily bouts of alcohol vapor exposure twice during the first week of vapor exposure and once during each subsequent week of vapor exposure. Plasma (5 μL) was used for the measurement of BALs using an Analox AM 1 analyzer (Analox Instruments, Stourbridge, UK). Vapor exposure occurred between 6 p.m. and 8 a.m. Following 7 weeks of CIE [24,46], rats were forcibly withdrawn from CIE and were euthanized at 5 time points during abstinence. During abstinence, animals in their cages were moved from the vapor chamber to normal housing at the Vivarium. These four time points were used for brain tissue processing for Figure 1 data: 1 d (n = 8), 7 d (n = 8), 21 d (n = 8), or 42 d (n = 8) (Figure 1). At each time point, ethanol-naïve rats (n = 4; housed in similar cages in the Vivarium) were euthanized (total n = 16). Neuroimaging data and immunoblotting data of oligodendroglial markers in the mPFC from these animals have been published elsewhere [47]. In the current study, mPFC tissue and DG tissue were used to determine changes in additional markers of synaptic plasticity. The rats in the fifth time point (3 d abstinence) experienced trace fear conditioning (TFC, see details below) before euthanasia for brain tissue processing. Age-matched controls were used for the 3 d time point animals (see details below). Data from these rats are used for Figure 2, Figure 3 and Figure 4. All animals were euthanized by rapid decapitation under isoflurane anesthesia for brain tissue processing.

### 2.3. Trace Fear Conditioning

*Apparatus*: Trace fear conditioning (TFC) was conducted in a set of four identical chambers housed within sound-attenuating boxes (Med Associates chambers connected to an AnyMaze interface and video tracking system). The floor was composed of stainless steel rods through which 0.5 mA shocks were delivered. Each chamber was illuminated by an overhead 7.5 W bulb and was connected to its own shock generator–scrambler. Ventilation fans provided constant background noise (~60 dB). Chambers were cleaned with a solution of quatricide disinfectant between animals. All training and testing sessions were conducted in the same chamber for each rat [21,23].

*Training*: Three days after CIE cessation, CIE males (CIE + TFC; n = 12) and age-matched control males (control + TFC, n = 9) were trained with TFC. Three days after CIE cessation, CIE males (CIE, n = 5) and age-matched control males (control, n = 7) were trained with sham conditioning (Figure 2). For training sessions of TFC, the animals received 5 series of CS–US presentations that occurred with varied intertrial intervals. The CS was a 30 s (second) tone cue (80 dB) and the US was a 1 s foot shock (0.5 mA). The CS and US were separated by an empty 45 s trace interval. The first CS presentation occurred following a 3 min (minute) baseline period and the final shock was followed by a 1 min post-shock period. For training sessions of sham conditioning, the control animals received a similar paradigm in similar boxes without any US (foot shock). 

*CS retrieval in fear context*: Twenty-four hours after training, animals were placed back in their original chambers for a 3 min baseline period after which they were presented with 5 CS-only presentations with each CS (30 s tone cue) separated by 45 s inter-interval. Immediately after the retrieval test, animals were returned to their home cages. One hour after the retrieval session, all animals were euthanized for brain tissue processing. TFC data were assessed as reported previously [23]. Freezing behavior during acquisition and retrieval is reported by subtracting baseline freezing from each rat to compute freezing during acquisition and retrieval [48]. TFC data, immunoblotting data, and Golgi–Cox data from the DG from these animals have been published elsewhere [23]. Additional data were collected from the DG and mPFC and have been reported in this study.

### 2.4. Western Blotting

Procedures optimized for measuring both phosphoproteins and total proteins were employed [49]. Tissue punches from the mPFC and dorsal hippocampal formation enriched in the DG (Figure 1b,c) from 500 μm thick sections were homogenized on ice by sonication in buffer (320 mM sucrose, 5 mM HEPES, 1 mM EGTA, 1 mM EDTA, 1% SDS, with Protease Inhibitor Cocktail and Phosphatase Inhibitor Cocktails II and III diluted 1:100; Sigma, Kawasaki City, Japan), heated at 100 °C for five minutes, and stored at −80 °C until the determination of protein concentration by a detergent-compatible Lowry method (Bio-Rad, Hercules, CA, USA). Whole lysates were used as we did not separate membrane and cytosolic fractions. Samples were mixed (1:1) with a Laemmli sample buffer containing β-mercaptoethanol. Each sample containing protein from one animal was run (20 μg per lane) on 10% SDS-PAGE gels (Bio-Rad) and transferred to polyvinylidene fluoride membranes (PVDF pore size 0.2 μm). Blots were blocked with 2.5% bovine serum albumin (for phosphoproteins) or 5% milk (*w*/*v*) in TBST (25 mM Tris–HCl (pH 7.4), 150 mM NaCl, and 0.1% Tween 20 (*v*/*v*)) for 16–20 h at 4 °C and were incubated with the primary antibody for 16–20 h at 4 °C: antibody to γ-aminobutyric acid (GABA)_A_ (1:500, PhosphoSolutions cat. no. 850-GA6, predicted molecular weight 60 kDa, observed band ~60 kDa), antibody to phosphorylated calcium/calmodulin-dependent protein kinase II (CaMKII), pCamKII Tyr-286 (1:200, Abcam cat. no. ab5683, Cambridge, UK, predicted molecular weight 50 kDa, observed band ~50 kDa), antibody to total (t)CamKII (1:200, Abcam cat. no. ab52476, predicted molecular weight 47 and 60 kDa, observed band ~47 and 60 kDa), antibody to total N-methyl-D-aspartate type glutamatergic receptors tGluN2A (1:200, Cell Signaling cat. no. 13185S, Danvers, MA, USA, predicted molecular weight 177 kDa, observed band ~170 kDa), antibody to tGluN2B (1:500, Santa Cruz Biotechnology cat. no. sc-9057, Dallas, TX, USA, predicted molecular weight 177 kDa, observed band ~180 kDa). Blots were then washed three times for 15 min in TBST and then appropriately incubated for 1 h at room temperature (24 °C) with horseradish peroxide-conjugated goat antibody to rabbit (1:500, BioRad) in TBST. After another three washes for 15 min with TBST, immunoreactivity was detected using SuperSignal West Dura chemiluminescence detection reagent (Thermo Scientific, Waltham, MA, USA) and collected using a digital imaging system (Azure Imager c600, VWR, Radnor, PA, USA) for Figure 1 data or HyBlot CL Autoradiography film (Denville Scientific, Bath, UK) and a Kodak film processor for Figure 4 data. For normalization purposes, membranes were incubated with 0.125% Coomassie stain for 5 min and washed three times for 5–10 min in destain solution [50,51]. Densitometry was performed using ImageStudio software (Li-Cor Biosciences, Lincoln, NE, USA). X-ray films were digitally scanned at 600 dpi resolution, then bands of interest were selected in identically sized selection boxes within the imaging program. The software Image J (version 1.45S, NIH) was then utilized to determine net intensity values. For total proteins, the signal value of the band of interest is expressed as a ratio of the corresponding Coomassie signal. For phosphoproteins, the signal value of the band of interest is expressed as a ratio of the corresponding total protein signal. This ratio of expression for each band is then expressed as a percent of the control included on the same blot.

### 2.5. Golgi–Cox Staining and Neuron Morphology Analysis

Brain tissue was submerged in Golgi–Cox solution A + B (FD Neurotechnologies Inc., Columbia, MD, USA) for 8 d at room temperature, followed by solution C for 4 d at room temperature, and stored at −80 °C until being processed for staining. Frozen brain tissue was coronally cut on a cryostat at 100 μm thick sections and stained with solution D + E and dehydrated according to the manufacturer’s instructions. Brains were coded before sectioning to ensure that the experimenters were blind to the treatments. To evaluate neuron morphology (pyramidal neurons or granule cell neurons), a Zeiss Axiophot microscope and a computer-based system (Neurolucida; Micro Bright Field, Williston, VT, USA) were used to generate three-dimensional (3D) neuron tracings that were subsequently visualized and analyzed using NeuroExplorer (Micro Bright Field). We selected neurons following four criteria: (1) the neuron was in the region of interest (prelimbic cortex or outer granule cell layer (GCL) of the superior or inferior blade of the dorsal DG), (2) the neuron was distinct from other neurons to allow for the identification of dendrites, (3) the neuron was not truncated or broken, and (4) the neuron exhibited dark, well-filled staining throughout, including spines. For each animal, 4–6 neurons from each brain region were traced at 40× magnification. For each reconstructed neuron, an estimate of dendritic complexity was obtained using the Sholl ring method. A 3D Sholl analysis was performed in which concentric spheres of increasing radius (starting sphere 10 μM and increasing in 20 μM increments; Figure 3a,d) were layered around the cell body until the dendrites were completely encompassed. The number of dendritic intersections at each increment was counted, and the results were expressed as the total intersections and the number of intersections per radial distance from the soma. Traced neurons (n = 4–6) from each rat were collapsed and the average from each rat was used for dendritic analyses.

### 2.6. Statistical Analyses

Parametric statistical analysis was used to analyze our datasets based on the assumption that our data fit a normal distribution and satisfy the sample size for adequate statistical power. Raw values of protein expression from Western blotting were analyzed by one-way or two-way ANOVA. For TFC analysis, the main dependent variable was the amount of time the rats spent engaged in freezing behavior. Freezing was defined as the absence of all movement except for that required for respiration. The average percent time spent freezing was calculated using the AnyMaze software (any-maze.com; StoeltingCo.com, Wood Dale, IL, USA). The AMI scoring parameters were chosen and freezing was analyzed as a percentage of each minute during the baseline, training, and testing sessions. None of the behaviors were hand-scored. Baseline freezing was evaluated as percent freezing, and freezing during training and testing sessions are indicated as the change in percent freezing from baseline freezing. Changes in freezing behavior during TFC acquisition were assessed by repeated measures two-way ANOVA (TFC session × treatment). Changes in freezing behavior during CS retrieval were analyzed by one-way ANOVA. Golgi–Cox data were analyzed using two-way ANOVA. Significant interaction or ANOVA was followed by post-hoc analysis using the Sidak multiple comparisons test. All graphs and statistical analyses were generated using Graph Pad version 7 for PC and *p* < 0.05 was considered statistically significant.

## 3. Results

**Figure 1:**
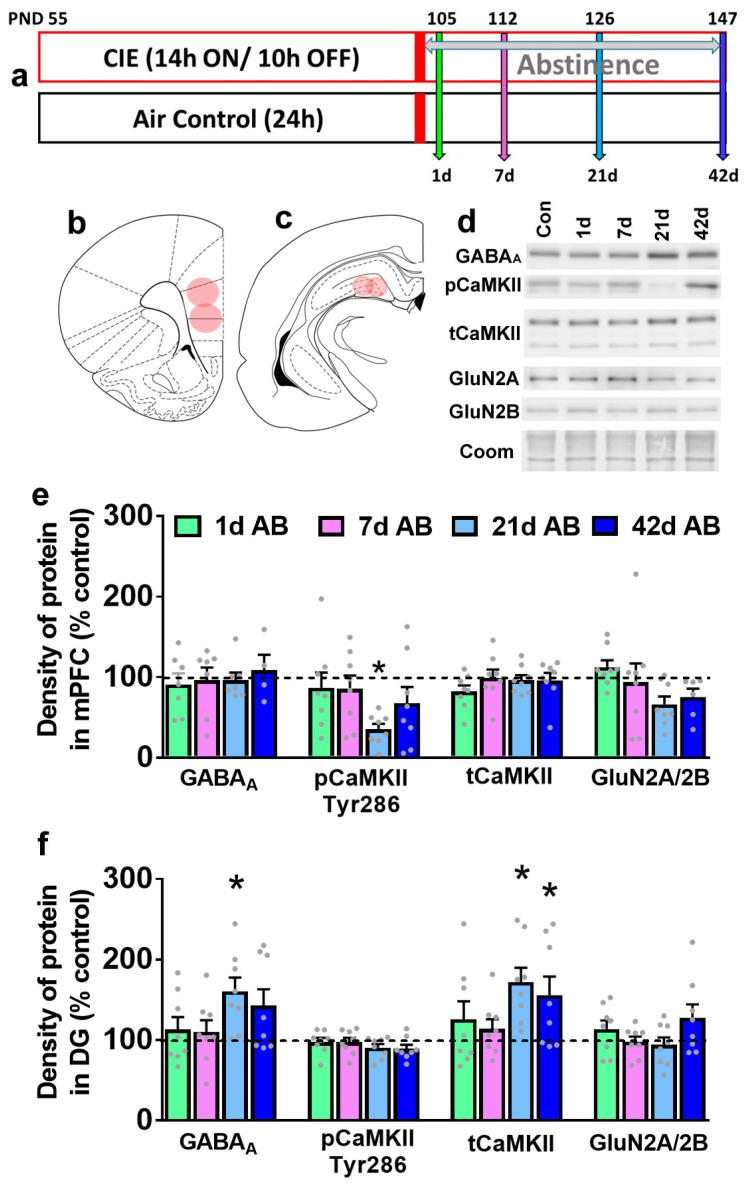
Abstinence-induced alterations in plasticity-related proteins in the mPFC and DG of the hippocampus. (**a**) Schematic of CIE weeks and time points during abstinence when brain tissue was harvested. (**b**,**c**) Coronal section of the rat brain indicating areas of tissue collected for immunoblotting analysis ((**b**), mPFC +3.7 from bregma; (**c**), DG −4.3 from bregma). (**d**) Representative immunoblots from all experimental groups in mPFC-enriched tissue. Coomassie was used as a loading control. (**e**,**f**) Quantitative data (mean ± SE) indicated as dot plots of GABA_A_, pCaMKII, tCaMKII, GluN2A/2B in the mPFC (**e**) and DG (**f**) indicated as percent change from the control condition. The control condition is indicated as a dashed line at 100%; * *p* < 0.05 vs. control condition. PND, postnatal day; AB, abstinence.

### 3.1. Abstinence Differentially Effects the Expression of Synaptic Plasticity-Associated Proteins over the Time Course in the mPFC and the DG

Expressions of GABA_A_, CaMKII, and GluN2A/2B ratio were determined at all time points in the mPFC and the DG. In the mPFC, one-way ANOVA did not detect any significant change in GABA_A_ (F(4,36) = 0.18, *p* = 0.9), tCaMKII expression (F(4,43) = 0.70, *p* = 0.5), or GluN2A/2B ratio (F(4,41) = 1.7, *p* = 0.15; Figure 1e). However, one-way ANOVA detected a significant change in pCaMKII (F(4,43) = 3.1, *p* = 0.02; Figure 1e). Post hoc analysis revealed lower levels of pCaMKII during abstinence at the 21 d time point compared to control rats. In the DG, one-way ANOVA did not detect any significant change in pCaMKII (F(4,43) = 1.4, *p* = 0.2) or GluN2A/2B ratio (F(4,41) = 1.8, *p* = 0.13; Figure 1f). However, one-way ANOVA detected a significant change in GABA_A_ (F(4,42) = 3.3, *p* = 0.02; Figure 1f) and tCaMKII expression (F(4,43) = 3.7, *p* = 0.01). Post hoc analysis revealed higher levels of GABA_A_ and tCaMKII during abstinence at the 21 d time point compared to control rats.

**Figure 2:**
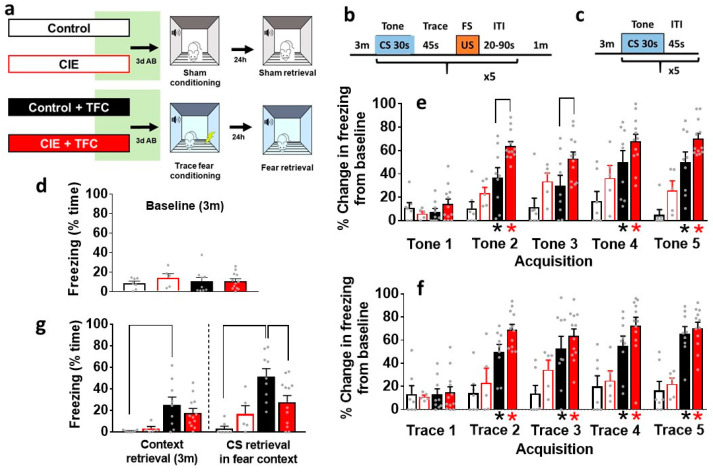
Abstinence alters the retrieval of trace fear memories. (**a**) Experimental groups with a timeline of CIE and TFC or sham conditioning. (**b**,**c**) Schematic of the TFC procedure, indicating details of the training during acquisition (**b**) and retrieval (**c**). Three days after the cessation of CIE, age-matched controls (CIE naïve) and CIE rats were trained on TFC (**e**,**f**), and 24 h after training, all animals were tested for CS retrieval (**g**). Freezing was monitored during the 3 min baseline session and during CS and trace intervals (**d**). (**d**) Quantitative data indicating freezing behavior during baseline. (**e**,**f**) Quantitative data indicating percent change in freezing from baseline during tone (**e**) and trace (**f**) periods. (**g**) Quantitative data indicating freezing behavior during retrieval. * *p* < 0.05 vs. control; * *p* < 0.05 vs. CIE. Data shown are represented as mean ± SEM. Control, n = 7; CIE, n = 5; control + TFC, n = 9; CIE + TFC, n = 12. CIE, chronic intermittent ethanol exposure; TFC, trace fear conditioning; CS, conditioned stimulus; US, unconditioned stimulus; FS, foot shock; ITI, intertrial interval; AB, abstinence; d, days; h, hours; m, minutes; s, seconds.

### 3.2. Freezing during Baseline Session

Control and CIE rats experienced sham sessions (control or CIE) or TFC (control + TFC or CIE + TFC) in the fear conditioning chambers. This experiment determined whether the CIE rats displayed differential freezing behavior to tone that was not conditioned to any fear behavior (foot shock). During the baseline period, animals in all groups showed equal freezing behavior, indicating that CIE rats did not display differential freezing behavior in response to tone in the fear conditioning chambers (Figure 2d). These data suggest that CIE rats did not reveal context generalization 3 d after the cessation of CIE. 

### 3.3. Freezing Behavior during Acquisition of TFC

We next evaluated the acquisition of TFC in control and CIE rats. Freezing data during acquisition were normalized to the baseline freezing behavior for each rat. During tone, repeated measures two-way ANOVA detected a group × freezing during CS interaction (F(12,145) = 2.6; *p* = 0.002), a main effect of groups (F(3,145) = 39.6; *p* < 0.0001), and freezing during CS (F(4,145) = 13; *p* < 0.0001). Post hoc analysis showed that the control + TFC rats had higher freezing behavior compared with their controls, and CIE + TFC rats had higher freezing behavior compared with CIE rats and control + TFC rats (Figure 2e). During trace, repeated measures two-way ANOVA detected a group × freezing during CS interaction (F(12,145) = 2.5; *p* = 0.004), a main effect of groups (F(3,145) = 39.6; *p* < 0.0001), and freezing during trace (F(4,145) = 11; *p* < 0.0001). Post hoc analysis showed that the control + TFC and CIE + TFC rats had higher freezing behavior compared with controls and CIE rats (Figure 2f).

### 3.4. Freezing Behavior during Context Retrieval and during CS Retrieval in Fear Context

Freezing behavior during context retrieval (3 min) and during CS retrieval were analyzed separately at 24 h post-TFC acquisition in all groups. Two-way ANOVA of context retrieval did not detect a significant CIE × TFC interaction or main effect of CIE; however, it detected a main effect of TFC (F(1,29) = 13; *p* = 0.009). Post hoc analysis showed that control + TFC rats had higher freezing behavior compared with control rats (*p* = 0.04; Figure 2g). The two-way ANOVA of CS retrieval detected a significant CIE × TFC interaction (F(1,29) = 7.3; *p* = 0.01) and a main effect of TFC (F(1,29) = 18; *p* = 0.002) without a main effect of CIE. Post hoc analysis showed that control + TFC rats had higher freezing behavior compared with all other groups (*p* = 0.04; Figure 2g).

### 3.5. Dendritic Arborization of Pyramidal Neurons in the mPFC and GCNs in the DG

**Figure 3:**
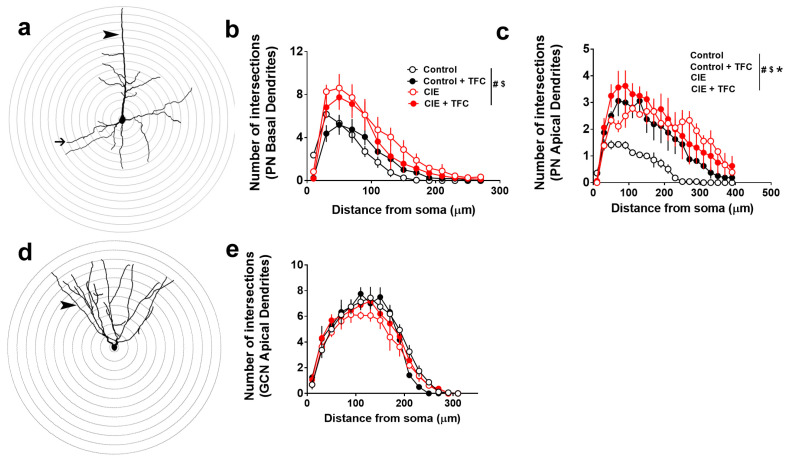
Abstinence and TFC produce distinct alterations in the structural plasticity of pyramidal neurons. (**a**) Example tracing of a pyramidal neuron with 3D sholl ring. Arrowhead points to apical dendrites and thin arrow points to basal dendrites. (**b**,**c**) Quantitative analysis of basal (**b**) and apical (**c**) dendrites plotted as the number of intersections on the x-axis and the distance from the soma on the y-axis. (**d**) Example tracing of a granule cell neuron in the DG with 3D sholl ring. Arrowhead points to apical dendrites. (**e**) Quantitative analysis of apical dendrites plotted as the number of intersections on the x-axis and the distance from the soma on the y-axis. PN, pyramidal neurons; GCN, granule cell neurons; TFC, trace fear conditioning. Signs after the vertical line in (**b**,**c**)—*p* < 0.05 (#) interaction, ($) main effect of distance, and (*) main effect of groups by ANOVA. Data shown are represented as mean ± SEM. Control, n = 4; CIE, n = 3; control + TFC, n = 4; CIE + TFC, n = 4.

Two-way ANOVA was performed to determine whether TFC in control and CIE rats differently affected the dendritic arborization of pyramidal neurons in the mPFC and GCNs in the DG (Figure 3). In the basal dendrites of the pyramidal neurons, repeated measures two-way ANOVA detected a group × distance from soma interaction (F(39,143) = 3; *p* < 0.001), a main effect of distance (F(13,143) = 115; *p* < 0.0001), and a trend towards a main effect of groups (F(3,11) = 2.8; *p* = 0.08). Post hoc analysis showed that CIE rats had higher arborization than controls, control + TFC rats had higher arborization than controls, and CIE + TFC rats had higher arborization than controls, CIE, and control + TFC rats (Figure 3b). In the apical dendrites of the pyramidal neurons, repeated measures two-way ANOVA detected a group × distance from soma interaction (F(57,209) = 1.5; *p* = 0.01), a main effect of distance (F(19,209) = 22.9; *p* < 0.0001), and a main effect of groups (F(3,11) = 14.5; *p* = 0.0004). Post hoc analysis showed that CIE, CIE + TFC, and control + TFC rats had higher arborization than controls, and CIE + TFC rats did not differ from CIE rats (Figure 3c). In the apical dendrites of the GCNs, repeated measures two-way ANOVA did not detect a group × distance from soma interaction (F(45,165) = 1; *p* = 0.4) or a main effect of groups (F(3,11) = 1; *p* = 0.4); however, it detected a main effect of distance (F(15,165) = 152; *p* < 0.0001; Figure 3e).

### 3.6. TFC Differentially Affects the Expression of GluN2A/2B Ratio in the mPFC and the DG

**Figure 4:**
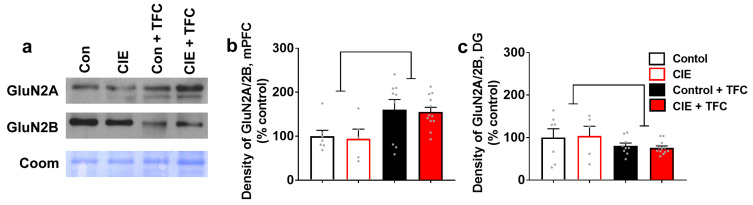
GluN2A/2B ratio is differentially altered in the mPFC and DG by TFC. (**a**) Representative immunoblots of GluN2A and GluN2B from all experimental groups in mPFC-enriched tissue. (**b**,**c**) Density of GluN2A/2B expression in mPFC (**b**) and DG (**c**). Main effect of TFC by ANOVA followed by post hoc tests is indicated with lines. Data shown are represented as mean ± SEM. Control, n = 7; CIE, n = 5; control + TFC, n = 9; CIE + TFC, n = 12.

The expression of GluN2A/2B ratio was determined in all groups (controls, CIE, controls + TFC, CIE + TFC) in the mPFC and the DG. In the mPFC, two-way ANOVA of the GluN2A/2B ratio did not detect a significant CIE × TFC interaction (F(1,29) = 0.0001; *p* = 0.9) or main effect of CIE (F(1,29) = 0.09; *p* = 0.76); however, it detected a main effect of TFC (F(1,29) = 11; *p* = 0.002; Figure 4b). In the DG, two-way ANOVA of the GluN2A/2B ratio did not detect a significant CIE × TFC interaction (F(1,29) = 0.1; *p* = 0.7) or main effect of CIE (F(1,29) = 0.0006; *p* = 0.9); however, it detected a main effect of TFC (F(1,29) = 3.5; *p* = 0.05; Figure 4c).

## 4. Discussion

The present results indicate that neuroadaptations in the mPFC and hippocampus exhibit clear distinctions and divergence during protracted abstinence in CIE rats. Notably, significant alterations in the expression of proteins linked to glutamatergic neurotransmission (CaMKII, GABA_A_) are evident at both 21 d and 42 d of abstinence. Interestingly, there were no alterations in these proteins during early abstinence (1 d, 7 d) in the mPFC and the hippocampus, suggesting other neuroadaptations during early abstinence after chronic ethanol experience [52]. CIE rats failed to show intact trace fear memories during early abstinence (3 d), implying that behavioral impairments were not predicted by alterations in the specific plasticity-related proteins in the brain regions studied. Interestingly, alterations in structural plasticity were observed in the pyramidal neurons in the mPFC during early abstinence, and these changes predicted behavioral deficits. CIE-induced hyperplasticity of pyramidal neurons was not attributed to changes in the expression of GluNs in the mPFC; therefore, other mechanisms could be assisting with the altered structural plasticity of mPFC pyramidal neurons. A limitation in the interpretation of these results is that the expression of GluNs in membrane-bound vs. cytosolic fractions was not evaluated and the activity states of these receptors were not explored, and these data sets could have provided additional mechanistic details on the effects of CIE-induced hyperplasticity and GluN signaling. Nevertheless, these findings demonstrate that there is a limited correlation between chronic ethanol-induced behavioral deficits and measures of plasticity-related proteins studied in the mPFC and hippocampus during early abstinence. The investigation of markers that demonstrate correlation, and importantly, causation, between behavior and neuroadaptations during early abstinence is warranted [53].

Previous studies from our laboratory have examined changes in oligodendrogenesis and myelination over several time points during abstinence from CIE (1 d to 42 d; [47]). Our results indicated deficits in oligodendrogenesis and myelination in the mPFC during abstinence from CIE, with a heightened rebound effect in oligodendrogenesis at 3 d and myelination at the 7 d time point [47,54,55]. Notably, increases in myelination at the 7 d time point are associated with gray matter abnormalities in the mPFC, supporting prior reports on the deficits in the structural and functional plasticity of neurons [43,47]. Additional analysis in the mPFC did not indicate concurrent changes in neuroimmune markers at the 7 d time point, indicating that alterations in oligodendrogenesis and myelination did not occur coincidentally to neuroimmune responses [47]. In the hippocampal DG, studies have indicated immune reactivity visualized as rebound microglial proliferation at the 3 d time point during abstinence [15,36,56]. Additional studies elucidated the altered structural and functional plasticity of neurons in the DG during late abstinence (>21 d), suggesting that neuroimmune activity in the DG during early abstinence may initiate some measures of neuroadaptations in the DG and that these adaptations could support relapse to ethanol-seeking behaviors [15,16,23,31]. We performed the current study to provide a more comprehensive analysis of the markers associated with neuronal plasticity in the mPFC and DG at a multitude of time points including 1 d, 7 d, 21 d, and 42 d into abstinence. We chose these time points as they capture the early abstinence and delayed abstinence time points in the animal model of moderate to severe AUD and because of the fact that the varied time points could predict the neurobiological changes associated with the negative affective state occurring during early and delayed abstinence [57]. We examined the changes in proteins associated with the glutamatergic transmission system as this system is perturbed following chronic ethanol treatment, with GluNs being particularly sensitive to ethanol exposure (reviewed in [58]). As GluNs are the primary source of glutamatergic transmission in the mPFC and the hippocampus, their regulation, expression, and subunit composition following CIE are critical to understanding the pathological plasticity subsequent to ethanol exposure. Previous studies of the GluNs have found the expression of the 2B subunit to be up-regulated following ethanol exposure in vitro [59,60] as well as in vivo in the hippocampus [31,37,40]. Of additional interest is the expression of the 2A subunit and the ratio of 2A to 2B. The 2A subunit, in the hippocampus, similar to the 2B subunit, has been found to be sensitive to ethanol with respect to expression [31,61,62]. More notable is that studies with comparative sensitivities to ethanol between the two subunits are rare, and in CIE rats, the ratio of 2A:2B is significantly higher during acute withdrawal when compared to protracted abstinence [31]. Increases in the ratio of 2A:2B in genetically modified animals have been linked to impaired hippocampal-sensitive cognitive and electrophysiological function [63], which could be occurring in CIE rats [16,42]. Our findings did not reveal any alterations in GluN2A or GluN2B levels or their ratio in the mPFC or the DG, supporting prior studies that have revealed alterations in these subunits during ethanol experience and not during abstinence [31,40,54]. Chronic ethanol experience also disrupts the delicate balance between GABA and glutamate in the hippocampus and PFC. Particularly interesting is the downregulation of GABA receptors in the hippocampus and PFC during CIE [30,37,64], and such neuroadaptations induced by ethanol could lead to behavioral deficits, including unregulated patterns of drinking and physical withdrawal behaviors [65]. Our studies did not reveal any significant changes in GABA_A_ receptors in the mPFC; however, there were increased levels of these receptors in the hippocampus, with significant effects at the 21 d time point. These findings suggest that hippocampal GABA_A_ receptors are more sensitive to the chronic effects of ethanol, and therefore have a rebound effect during abstinence to facilitate relapse to ethanol-seeking behaviors [66]. We investigated the effects of abstinence on the activity of CaMKII, as it is influenced by synaptic plasticity [67,68]. The finding of reduced activity of CaMKII in the mPFC and enhanced expression of the protein in the hippocampal DG is important because it may coincide with dysregulated glutamate levels in these brain regions during protracted abstinence [69]. Taken together, these findings of distinct alterations in plasticity-related proteins in the mPFC and hippocampal DG suggest that future research should further characterize the implications of such opposing alterations in regulating the negative affective state during abstinence and investigate if blocking such dysregulation reduces relapse to drinking behavior.

The formation and expression of fear memories in a time-limited manner depend on a functional and intact mPFC and hippocampus [70,71,72,73]. Mechanistic studies demonstrate that the dorsal hippocampus, specifically the DG, and the mPFC are involved in the acquisition, consolidation, and expression of TFC [73,74,75,76,77,78]. In the context of AUD, short-term and prolonged ethanol exposure does not alter the acquisition of fear responses in delayed fear conditioning and TFC [17,18,19,20,21,23,79,80,81]; however, it produces deficits in CS retrieval, seen as reduced freezing or amnesic effects in response to the CS in a novel context [17,18,19,20,21,23]. The impaired and amnesic effect in ethanol-experienced animals could result from neuroplastic and neuroadaptive changes in the mPFC and hippocampal DG [21,23,78,80,82,83]. For example, several neuroplastic and neuroadaptive changes are seen in the mPFC and hippocampus after chronic ethanol experience (reviewed in [33,84,85,86,87,88]). In the hippocampal DG, studies have indicated immune reactivity visualized as rebound microglial proliferation at the 3 d time point during abstinence [15,36,56]. Such neuroplastic changes could contribute to impairments in emotional memories, particularly fear retrieval. We report that CIE rats showed reduced retrieval of fear memories during early abstinence (3 d time point). We therefore examined whether reduced retrieval was associated with the altered structural plasticity of neurons in the mPFC and the hippocampal DG. We sought to examine the effects of CIE and TFC in CIE rats on the structural plasticity of pyramidal neurons in the mPFC and GCNs within the DG of the hippocampus. The analysis of Golgi–Cox labeled cells revealed a significant increase in the dendritic arborization of the basal dendrites of the mPFC neurons under CIE and TFC + CIE conditions, indicating that the increases in arborization were a consequence of CIE and not TFC behavior. Furthermore, increases in the arborization of mPFC neurons were also evident in the apical dendrites, with TFC increasing dendritic complexity under naïve control conditions. This effect was not evident in CIE rats, where dendritic complexity was increased under CIE and TFC + CIE conditions. These findings indicate that apical dendrites showed tolerance to TFC’s effect on the structural plasticity of mPFC neurons in CIE rats and that this effect was associated with reduced freezing during CS retrieval. This is important because chronic ethanol exposure increases the dendritic arborization and dendritic spine density of mPFC neurons, indicating dendritic remodeling during CIE [43,49,54,80]. Although alterations in neuron structure are not required for alterations in neuron function [89], changes in the dendritic structure of mPFC neurons have been correlated with changes in the functional plasticity of mPFC neurons during alcohol exposure [43,45,80]. This association between alterations in the structure and function of mPFC neurons has been extended into abstinence, as 21 d abstinence post-CIE did not abolish the structural and functional changes seen during alcohol exposure [43,54]. Furthermore, CIE induces emotional memory deficits dependent on the mPFC which persist into abstinence [80,90], and these behavioral impairments could be attributable to the maladaptive plasticity of pyramidal neurons. For example, the apical dendrites of the pyramidal neurons in the mPFC receive afferent inputs from the basolateral nucleus of the amygdala, which make connections at approximately 50–70 μm from the soma [91,92]. Therefore, a blunting of dendritic responses to TFC in apical dendrites during abstinence may suggest altered synaptic transmission from the amygdala, thus promoting emotional memory deficits, including reduced fear retrieval [93,94]. Surprisingly, no changes were noted in GCNs from the DG, indicating that DG GCNs were not affected by CIE or TFC at the 3 d time point and that other neuronal cell types in the hippocampus may be playing a role in assisting with the behavioral deficits [23,31].

In parallel, we determined whether proteins influencing structural plasticity, including GluN2A and GluN2B, were altered. This is because studies have demonstrated a mechanistic role of GluNs in acquisition and CS retrieval when subjects were trained in a TFC paradigm [95,96,97,98]. For example, the blockade of GluN receptors prior to training disrupts the consolidation of fear memories [99], and similarly, the genetic deletion of hippocampal GluNs disrupts TFC [97]. The GluN2A and GluN2B subunits mark the principal GluN subtypes, and the ratio of GluN2A/2B determines the qualitative and functional properties of neurons and affects the expression of fear memories [63,98,100,101,102]. Our findings demonstrate that TFC enhances the GluN2A/2B ratio under control and CIE conditions in the mPFC and reduces the GluN2A/2B ratio under these conditions in the DG. Since there were no differences between control and CIE conditions in the ratio of GluN2A/2B at the 3 d time point, and there were no differences in the ratio between the control and CIE conditions after TFC, in the mPFC, we speculate that there are other plasticity-related mechanisms contributing to the blunting or tolerance-like effect in the structural plasticity of pyramidal neurons. Taken together, these findings demonstrate brain region-specific alterations in the structural plasticity of neurons under CIE and TFC conditions whose expression correlates with the consolidation of fear memories.

## Data Availability

Data will be made available upon request due to ethical restriction.
